# Treatment of Highland Frogs from the Two-Legged Stage with Homeopathically Prepared Thyroxin (10^-11^–10^-21^)

**DOI:** 10.1100/tsw.2008.58

**Published:** 2008-04-20

**Authors:** Gerhard Lingg, P. Christian Endler, Michael Frass, Harald Lothaller

**Affiliations:** ^1^ Interuniversity College for Health and Development Graz/Castle of Seggau, Austria; ^2^ University of Graz, Austria

**Keywords:** amphibian, hormone, thyroxin, homeopathic dilution, Q-dilution, hormesis

## Abstract

The influence of moderately diluted, agitated, i.e., homeopathically prepared, thyroxin solutions (10^-11^–10^-12^, final concentration in the basin water 0.6 × 10^-15^ − 0.6 × 10^-25^ parts by weight after the first application) on metamorphosis in highland *Rana temporaria* from the two-legged stage was studied. In accordance with the homeopathic idea of effects of specially prepared dilutions being inverse to those of their mother substances, animals were treated either with thyroxin 10^-11^–10^-21^ or analogously prepared blank solution (water). Development was monitored by documenting the number of animals that had entered the four-legged stage. It has been found that animals treated with the thyroxin solutions metamorphosed more slowly than the control animals, i.e., the effect of the homeopathically prepared thyroxin was opposed to the usual effect of molecular thyroxin. The number of test animals that reached the four-legged stage at defined points in time was smaller (2–13.5%) in the group treated with homeopathically prepared thyroxin at the points in time, compared to control. The results in this study sustain the previous multiresearcher findings that show that diluted homeopathically prepared thyroxin is able to slow down metamorphosis of *R. temporaria*.

## INTRODUCTION

Previous experiments performed in five independent laboratories in Austria and the Netherlands have shown that a homeopathically prepared dilution of thyroxin $10^{-30}$ (“decimal dilution”, final concentration in the basin water $10^{-35}$) was used to slow down metamorphosis from the two- to four-legged stage in highland *Rana temporaria* (3–11%; $p < 0.001$). Thyroxin $10^{-30}$ was compared to analogously prepared solvent water $10^{-30}$; number of animals = 1,620 per group[1,2,3,4].

Further studies on thyroxin $10^{-30}$ were discussed elsewhere, namely analogous experiments with animals from lowland biotopes[5], experiments with highland[3,4] and lowland[5] animals pretreated with molecular thyroxin, and experiments with lowland animals treated from the spawn stage on[6].

For the experiment presented here, we used the same setup as for the initial multilaboratory study[1,2,3,4], but a special preparation process of the homeopathic drug involving dilution steps of 1: 50,000 and leading to thyroxin $10^{-11} – 10^{-21}$ was used.

## METHODS

The experiments were carried out by Lingg in his laboratory at Bregenz, Austria. The study was planned by Lingg and Endler. Application of the homeopathically prepared thyroxin or solvent was done blind.

### Animals, Staging, Water. and Further Laboratory Conditions

*Rana temporaria* larvae were taken from highland pools in the Austrian Alps, approximately 1,500 m above sea level. The starting stage for treatment was defined as the point at which the hind legs of the two-legged tadpoles are straddled, such that one can only just see through the triangle formed by thigh, shank, and tail. This point of development occurs during Gosner's stage 31[7, p. 45]. The tadpoles were observed until the forelegs would break through the skin and the animals thus entered the four-legged stage. Basins contained 7.5 l of dwell water each; water was not refreshed during the experiment.

In experiment 1, 40 animals were always allotted to each of a total of eight white plastic basins according to a random procedure. In experiments 2 and 3, 25 animals were always allotted. Indirect natural light was used. Room temperature was 20 ± 2°C. The tadpoles were fed with blanched greens (lettuce) *ad libitum*. Experiments were carried out in August and September 2007.

### Preparation and Administration of Test Solutions

One group of two-legged animals were treated with the homeopathically prepared test dilutions of tetraiodothyronine sodium pentahydrate (L-thyroxine, $T_4$, Sigma), while the other one was treated with the analogously prepared solvent. Dilutions Q1 (= $2 \times 10^{-11}$ parts by weight), Q2 (= $4 \times 10^{-16}$), and Q3 (= $8 \times 10^{-21}$) were prepared according to standardized instructions[8]. These involved dilution steps of 1:50,000 and vigorous agitation between steps. Preparation of test and control substances was done by K. Leisser, Homeocur ltd, Retz, Austria.

A quantity of 0.25 ml of probe dilution (test or control) was added per 7.5 l of basin water at intervals of 8 h. Thus, final concentration in the basin water would be $0.6 \times 10^{-15}$ after first application of Q1, $0.6 \times 10^{-20}$ of Q2, and $0.6 \times 10^{-25}$ of Q3. At day 1, Q1 was used, at day 2, Q2 (etc., see Table 1**).**

**TABLE 1**
**Application Scheme of Test and Control Dilutions (for explanation, see text)**

| Day 1 22 h | Day 2 06 h | Day 2 14 h | Day 2 22 h | Day 3 06 h | Day 3 14 h | Day 3 22 h | Day 4 06 h | Day 4 14 h | Day 4 22 h | Day 5 06 h | Day 5 14 h | Day 5 22 h |
|---|---|---|---|---|---|---|---|---|---|---|---|---|
| Q1 | Q2 | Q2 | Q2 | Q3 | Q3 | Q3 | Q1 | Q1 | Q1 | Q2 | Q2 | Q2 |

### Ascertaining and Evaluating the Data

Following a suggestion by R. Lüdtke, Institut für Medizinische Informationsverarbeitung Tübingen University[5], the points in time when the numbers of water control animals that had reached the fourlegged stage were closest to the 10th, 20th, 30th, 40th, 50th, 60th, and 70th percentiles were defined as reference points. The time interval between reference points normally was 8 or 16 h. In those cases, when values of water control animals were, for example, 6% in one point in time and 14% in the following, we decided to use the arithmetic mean (10%) both for water and control values. In this way, it was possible to aggregate, for each of the reference points, the cumulative frequency of animals treated with control or test solution having reached the four-legged stage. Aggregate values obtained at the reference points for each of the types of treatment were analyzed by Chi-square tests using four-field tables with aggregate frequencies of two- or three-legged animals as complement. According to a Bonferroni correction for multiple measurements (i.e., 7), $p$ values would have to be <0.007 to signify statistical significance.

Different statistical methods had been discussed in connection with the amphibian model previously, such as variance analysis, t-test, survival analysis, proportional hazards model, and logistic regression[5]. These usually lead to comparable results but need larger numbers of basins in one and the same experiment. Furthermore, depending on differences in the overall duration of experiments, standard deviation (SD) is usually contorted when experiments from different laboratories are pooled. Also, in order to make results comparable to those of previous publications, we restricted ourselves to the (comparatively rough and estimative) Chi-square test.

## RESULTS

There were 260 animals treated with thyroxin Q dilutions and 260 animals with analogously prepared water. As shown in Fig. 1 and Table 2, animals treated with the test solution (black squares) metamorphosed more slowly than the control animals (white squares) at most points in time (2–13.5%). The $p$ values were significant at three points in time, out of which one was statistically significant after Bonferroni correction. SD was about 7–10%.

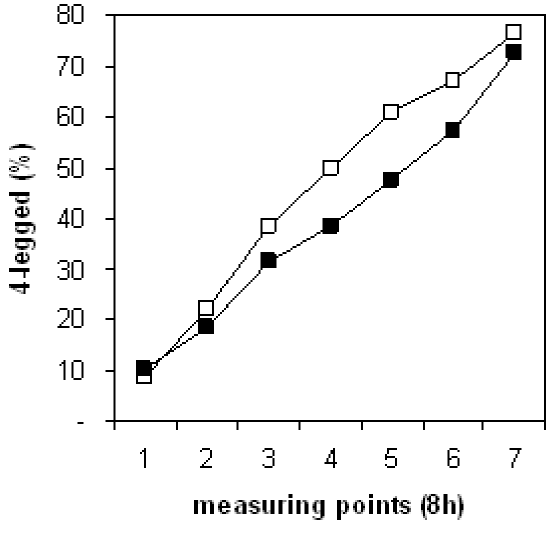

**FIGURE 1.** The effect of thyroxin $10^{-11}$ – $10^{-21}$ ("Q" dilutions 1, 2, and 3) on highland *R. temporaria.* Ordinate = cumulative frequency of four-legged tadpoles (N). Abscissa = points in time. Black squares = frequencies of animals treated with homeopathically prepared thyroxin; white squares = animals treated with analogously prepared water. For further details, see text.

**TABLE 2**
**The Effect of Thyroxin $10^{-11}$ – $10^{-21}$**

| Measuring Point | 1 | 2 | 3 | 4 | 5 | 6 | 7 |
|---|---|---|---|---|---|---|---|
| N water Q – animals | 22 | 57 | 100 | 130 | 158 | 174 | 199 |
| N thyroxin Q – animals | 27 | 48 | 82 | 100 | 123 | 149 | 189 |
| P | 0.53 | 0.35 | 0.13 | 0.01 | 0.003 | 0.03 | 0.39 |
| Sign. without corr. | — | | — | ** | ** | * | — |
| Sign. Bonferroni corr. | — | — | — | — | ** | — | — |

N = Numbers of four-legged animals at the measuring points; P = *p* values (Chi-square tests); Sign. = significance without correction and Bonferroni corrected. * = significant; ** = highly significant

Values for thyroxin Q – animals were below those of water Q – animals in each of the subexperiments including 50 + 50, 50 + 50, and 160 + 160 animals.

Frequencies of animals treated with homeopathically prepared thyroxin reached the values of control animals with a delay of about 0.29–1.39 intervals between points in time (= 2–11 h).

Thus, in accordance with our previous studies, the effect of the homeopathically prepared thyroxin was opposed to the usual stimulating effect of molecular thyroxin.

## DISCUSSION

From our studies[1,2,3,4,5,6,7,9], there appears to be a relationship between the effect of homeopathically prepared thyroxin and an elevated thyroxin level in the animals during metamorphosis.

Thyroxin diluted in steps of 1:10 (“decimal dilution”) up to $10^{-30}$ led to a slowing down of metamorphosis of about 3–11%[1,2,3,4]. Thyroxin diluted in steps of 1:50,000 (“Q”-dilution) up to $10^{-11}$ – $10^{-21}$ also led to a slowing down of 2–13.5% (the study presented here).

From a theoretical point of view, it seems interesting that information from the original thyroxin molecules may have been “imprinted” in water by input of kinetic energy in a sequence of only a few steps of dilution 1:50,000 plus vigorous agitation with a comparable result by a much longer sequence of steps of 1:10. It goes without saying that further investigation should concern both “decimal” dilutions in the range of $10^{-11}$ – $10^{-21}$ as well as “Q” dilutions in the range of $10^{-30}$.

Additionally, the so-called “hormesis” effect of moderate molecular dilutions[7, p. 5ff], prepared without “homeopathic” agitation phases, could be investigated with the amphibian model.

## References

1. Endler, P.C., Pongratz, W., Van Wijk, R., Kastberger, G., and Haidvogl, M. (1991) Effects of highly diluted succussed thyroxin on metamorphosis of highland frogs. *Berlin J. Res. Hom.* **1**, 151–160.
2. Zausner, C., Lassnig, H., Endler, P.C., Scherer, W., Haidvogl, M., Frass, M., Kastberger, G., and Lüdtke, R. (2002) Die Wirkung von "homöopathisch" zubereitetem Thyroxin auf die Metamorphose von Hochlandamphibien - Ergebnisse einer multizentrischen Kontrollstudie. *Perfusion* **17**, 268–276.
3. Welles, S.U., Endler, P.C., Scherer-Pongratz, W., Suanjak-Traidl, E., Weber, S., Spranger, H., Frass, M., and Lothaller, H. (2007) Pretreatment with thyroxin 10(-8) and the effect of homeopathically prepared thyroxin 10(-30) on highland frogs--a multi-researcher study. *Forsch. Komplement. Med.* **14**, 353–357. http://content.karger.com/ProdukteDB/produkte.asp?doi=111540
4. Weber, S., Endler, P.C., Welles, S.U., Suanjak-Traidl, E., Scherer-Pongratz, W., Frass, M., Spranger, H., Peithner, G., and Lothaller, H. (2008) The effect of homeopathically prepared thyroxine on highland frogs: influence of electromagnetic fields. *Homeopathy* **97**, 3–9.

5. Endler, P.C., Lüdtke, R., Heckmann, C., Zausner, C., Lassing, H., Scherer-Pongratz, W., Haidvogl, M., and Frass, M. (2003) Pretreatment with thyroxin ($10^{-8}$ parts by weight) enhances a "curative" effect of homeopathically prepared thyroxin ($10^{-13}$) on lowland frogs. *Forsch. Komplementarmed. Klass. Naturheilkd.***10**, 137–142.
6. Graunke, H., Endler, P.C., Scherer-Pongratz, W., Frass, M., and Lothaller, H. (2007) Treatment of lowland frogs from the spawn stage with homeopathically prepared thyroxin (10(-30)). *TheScientificWorldJOURNAL* **7**, 1697–1702.
7. Endler, P.C. and Schulte, J. (1998) *Ultra High Dilution. Physiology and Physics*. Kluwer Academic, Dortrecht.
8. Deutscher Apotheker Verlag (2007) *Homöopathishes Arzneibuch 2006. Amtliche Ausgabe.* Deutscher Apotheker Verlag, Stuttgart.
9. Endler, P.C., Pongratz, W., Smith, C.W., and Schulte, J. (1995) Non-molecular information transfer from thyroxin to frogs with regard to homeopathic toxicology. *Vet. Hum. Toxicol*. **37**, 259–260.
